# Timing for Surgical Stabilization with K-wires after Open Fractures of Proximal and Middle Phalangeal Shaft

**DOI:** 10.1038/s41598-017-11918-2

**Published:** 2017-09-12

**Authors:** Yanchun Gao, Qiyang Wang, Hongyi Zhu, Zhengyu Xu

**Affiliations:** Department of Orthopaedic Surgery, Shanghai Jiaotong University Affiliated Sixth People’s Hospital, Shanghai, 200233 China

## Abstract

The optimal timing for surgical stabilization after open fractures of proximal and middle phalangeal shaft remained unclear. Total 147 patients with single open fracture in proximal or middle phalangeal shaft (arrived within 8 hours) who received K-wire fixation from June 2012 to June 2015 were included for analysis. The timing for surgical stabilization of fractures (immediate or delayed) was decided according to the surgeons’ preferences. The Michigan hand outcomes questionnaire (MHQ) scores, grip strength and total active motion (TAM) one year after the initial surgery were similar between the two groups. There was no significant difference in the incidence of tenosynovitis, bone nonunion. The overall infection rate in immediate fixation group was slightly but not significantly higher compared with the delayed fixation group (29.2% versus 20.7% *P* = 0.212). However, patients with both palmar and dorsal wounds who received immediate fixation had much higher infection rate compared with delayed fixation (52.6% versus 22.7%, *P* = 0.047). The immediate fixation could reduce costs and the period of hospitalization. Open fractures with both palmar and dorsal wounds should be treated with delayed fixation of K-wires otherwise stabilized immediately after injury.

## Introduction

Phalangeal fractures are a common type of fractures which can lead to hand function impairment. Among 1.5 million hand and forearm fractures, 23% were phalangeal fractures^1^. Phalangeal fractures might result in unsatisfactory outcomes possibly because these phalangeal fractures are regarded as trivial injuries^2^. For stable fractures of proximal and middle phalangeal shaft, nonsurgical management is the first-line therapy^3^. For unstable or complex ones, surgical stabilization shows advantages in long-term prognosis^4^. A range of surgical techniques have been described for the treatment of proximal and middle phalangeal fractures. Currently used techniques included crossed K-wires^5^, lag screw^6^, locking plate^7^ fixation and K-wire fixation is the reference treatment for phalangeal fractures^8^.

There was, to the best of our knowledge, no evidence showing the optimal timing for surgical stabilization after open fractures of proximal and middle phalangeal shaft. It was possible that immediate fixation could increase the rate of infection. However, delayed surgery would lead to increased costs and prolonged hospitalization. In this study, we compared the outcomes between immediate and delayed K-wire fixation after open fractures of proximal and middle phalangeal shaft.

## Methods

The study was approved by the Ethics Committee of Shanghai Jiaotong University Affiliated Sixth People’s Hospital. Informed consent was obtained from all participants. All study methods were in accordance with the Declaration of Helsinki.

Patients with single open fracture in proximal or middle phalangeal shaft (arrived within 8 hours) who received K-wire fixation were included for analysis from June 2012 to June 2015. The K-wires were removed 8 weeks after the fixation. Inclusion criteria were completion of at least one year follow-up, age ranged from 18 to 60. Patients with any of the following criteria were excluded: two or more fractures in upper limber, any injuries beyond the finger with fracture, injuries on artery.

After initial evaluation, all open wounds were irrigated with saline solution and dressed with sterile gauze. The patients were routinely given tetanus prophylaxis. All patients received cephalosporin antibiotics immediately in the emergency department and for three consecutive days. All patients received irrigation and debridement in the operative theater of emergency department. In this study, no patients had flexor tendon injuries. After irrigation and debridement, tendons and nerves were repaired if injured. Due to the lack of evidence, the timing for K-wire fixation was based on surgeons’ preferences. Sixty-five patients received K-wire fixation immediately after injury in the emergency department while 82 patients were admitted to hospital and received delayed fixation at least three days after injury (the fixation of fracture might be further delayed if infection occurred). All surgeries in this study were conducted by specialists of hand surgery. Active mobilization was encouraged immediately after surgery.

McLain modified the Gustilo-Anderson classification and described type I fractures as clean wounds smaller than 1 cm with no crush of skin or comminution of bone. Type II fractures were those with clean wounds greater than 1 cm with no crush of skin or comminution of bone. Type III injuries had wounds with contamination, soft tissue crush, or comminuted fractures^9^.

Demographic parameters including age and gender were recorded for each group. Assessments were conducted at one year after surgery by a trained researcher who was unaware of the details of the patients’ treatment. Total active motion (TAM) is the sum of angles formed by MP, PIP, and DIP joints in maximum active flexion^10^. Michigan hand outcomes questionnaire (MHQ) was used for Clinical assessment^11^. The MHQ includes 6 distinct subscales: overall hand function, activities of daily living, pain (reversed), work performance, aesthetics, and patient satisfaction. The MHQ total score is obtained by averaging the scores for all 6 subscales. The score ranges from 0 to 100 with a higher score indicating better hand performance. The direct costs included fees for surgery, hospitalization and outpatient services. The costs were calculated according the receipts of hospital.

Statistical analysis was performed using SPSS 22.0 software. A *P* value of < 0.05 was considered to be statistically significant. Data were presented as mean ± standard deviation. Student’s t and chi-square tests were used to compare numeric and nonnumeric variables respectively.

## Results

Total 147 patients were included for analysis in our study. All of the surgeons were specialists of hand surgery. Patients were divided into two groups based on the timing of internal fixation. The average of follow-up period was 29 months (12 to 48 months). In our series, 104 of 147 cases were male. The average age of this series was 37.3 ± 12.0 (18 to 60). The right hand was affected in 79, the left hand in 68 cases. Statistically, the data in Table 1 showed no significant difference of demographics between two groups.

**Table 1:** Patient features.

	Immediate fixation	Delayed fixation	*P* Value
Male	45 (69.2%)	59 (72.0%)	0.719
Female	20 (30.8%)	23 (28.0%)	
Left	29 (44.6%)	39 (47.6%)	0.588
Right	36 (55.4%)	43 (52.4%)	
Age	38.7 ± 12.4	36.2 ± 11.6	0.207
Extensor tendon rupture	13 (20%)	18 (22.0%)	0.773
Injured finger			
Index	18 (27.7%)	28 (34.1%)	0.866
Long	18 (27.7%)	20 (24.3%)	
Ring	15 (23.1%)	18 (22.0%)	
Small	14 (21.5%)	16 (19.5%)	
Wound sites			
Dorsal	31 (47.7%)	42 (51.2%)	0.911
Palmar	15 (23.1%)	18 (22.0%)	
Both	19 (29.2%)	22 (26.8%)	
Modified Gustilo-Anderson fracture type			
I	13 (20.0%)	17 (20.7%)	0.980
II	20 (30.8%)	24 (29.3%)	
III	32 (49.2%)	41 (50.0%)	

The clinical outcomes one year after treatment were shown in Table 2. It was conceivable that there was no significant difference between the two groups in TAM (232.9 ± 22.5 versus 227.4 ± 33.5 degrees, *P* = 0.447), grip strength (92.8 ± 5.5 versus 93.8 ± 8.6,*P* = 0.253) and MHQ scores (91.0 ± 9.44 versus 90.8 ± 7.91, *P* = 0.302).

**Table 2:** Clinical outcomes.

	Immediate fixation	Delayed fixation	*P* Value
TAM (degree)	232.9 ± 22.5	227.4 ± 33.5	0.447
Grip strength (%)	92.8 ± 5.5	93.8 ± 8.6	0.253
MHQ	91.0 ± 9.44	90.8 ± 7.91	0.302

The rate of complications in both group were summarized in Table 3. There was no significant difference in the incidence of tenosynovitis, bone nonunion. The infection rate in immediate fixation group was slightly but not significantly higher compared with the delayed fixation group (29.2% versus 20.7% *P* = 0.212). Interestingly, we found the infection rate of patients with both palmar and dorsal wounds who received immediate fixation was much higher than compared with delayed fixation.

**Table 3:** Complication.

	Immediate fixation	Delayed fixation	*P* Value
Overall Infection	19(29.2%)	17 (20.7%)	0.234
Infection (grouped by wound sites)			
Dorsal	6 (19.4%)	8 (19.0%)	0.974
Palmar	3 (20.0%)	4 (22.2%)	0.890
Both	10 (52.6%)	5 (22.7%)	0.047
Infection (grouped by fracture type)			
I	2 (15.4%)	1 (5.9%)	0.390
II	5 (25.0%)	4 (16.7%)	0.495
III	12 (37.5%)	12 (29.3%)	0.458
Tenosynovitis	2 (3.1%)	3 (3.7%)	0.852
Nonunion	0 (0%)	1 (1.2%)	0.372

As we assumed and shown in Table 4, the immediate fixation could reduce costs (2712 ± 519versus 13019 ± 3536 CNY, *P* < 0.001) and hospitalization (0.24 ± 0.07 versus 9.21 ± 2.17 days, *P* < 0.001). The sick leave periods were similar between the two groups.

**Table 4:** Economic analysis.

	Immediate fixation	Delayed fixation	*P* Value
Direct cost (CNY)	2712 ± 519	13019 ± 3536	<0.001
Hospitalization (days)	0.24 ± 0.07	9.21 ± 2.17	<0.001
Sick leave (weeks)	12.89 ± 4.91	14.3 ± 4.45	0.348

CNY = China Yuan.

## Discussion

Infection, as the most common complication in open phalangeal fractures, was reported with an incidence from 2% to 30%. The presence of infection was shown to be highly correlated with functional outcome^9, 12, 13^. The cornerstone of open fracture management is appropriate irrigation and debridement to prevent infection. Insertion of implants including K-wires immediately after injury might increase the infection rate by hampering the pathogen elimination. However, there was no previous study investigating this issue.

Consistent with previous studies, infection rate increased along with the grades of Gustilo-Anderson classification. However, Gustilo-Anderson showed poor value in fixation timing decision-making. Instead, our study revealed that immediate fixation was inappropriate for open fractures with both palmar and dorsal wounds. Delayed fixation showed a lower infection rate for this type compared with immediate stabilization. For open fracture with single palmar or dorsal wound, immediate fixation was superior with similar outcomes and lower costs.

## Conclusion

Open fractures with both palmar and dorsal wounds should be treated with delayed fixation of K-wires otherwise stabilized immediately after injury.
